# The motor prodromes of parkinson’s disease: from bedside observation to large-scale application

**DOI:** 10.1007/s00415-019-09642-0

**Published:** 2019-12-04

**Authors:** C. Simonet, A. Schrag, A. J. Lees, A. J. Noyce

**Affiliations:** 1 Preventive Neurology Unit, Wolfson Institute of Preventive Medicine, Queen Mary University of London, London, UK; 2 Department of Clinical and Movement Neurosciences, Institute of Neurology, University College London, London, UK; 3 Reta Lila Weston Institute of Neurological Studies, University College London, London, UK

**Keywords:** Parkinson’s disease, Prodromal phase, Subtle motor signs, Quantitative motor analysis, Population studies

## Abstract

There is sufficient evidence that the pathological process that causes Parkinson’s disease begins years before the clinical diagnosis is made. Over the last 15 years, there has been much interest in the existence of a prodrome in some patients, with a particular focus on non-motor symptoms such as reduced sense of smell, REM-sleep disorder, depression, and constipation. Given that the diagnostic criteria for Parkinson’s disease depends on the presence of bradykinesia, it is somewhat surprising that there has been much less research into the possibility of subtle motor dysfunction as a pre-diagnostic pointer. This review will focus on early motor features and provide some advice on how to detect and measure them.

## Introduction

The diagnosis of Parkinson´s disease (PD) relies on operational clinical diagnostic criteria first proposed in 1992 and based on clinico-pathological correlation [1]. They require the presence of bradykinesia, a progressive reduction in the speed and amplitude of repetitive self-willed movements (sequence effect), plus at least one further parkinsonian sign from rigidity, rest tremor, and/or postural instability [1, 2]. By the time an individual fulfils clinical diagnostic criteria, at least 40% of nigral dopaminergic neurons have been lost [3].

A large number of longitudinal studies support the link between several symptoms such as hyposmia, REM-sleep behaviour disorder (RBD), constipation, and depression, with the subsequent emergence of PD [4]. These non-motor clinical features do not necessarily occur in synchrony and little is known about their natural course [5], or whether they progress at all. Somewhat surprisingly, much less attention has been paid to the subtle motor dysfunction that precedes overt bradykinesia and a diagnosis of PD [6].

## Towards early and timely diagnosis

Several factors may influence a delay in diagnosis even when motor signs are evident. These include available and equitable access to healthcare and health professionals, the clinical experience and expertise of those health professional, as well as patient comorbidity that may mask or confound the clinical impression. In addition to these challenges, there are patient-related factors that may lead to a delay in recognition of the early motor features of PD. Many patients with PD seem to have a visuo-perceptual agnosia to their motor handicap, or attribute changes to tiredness or aging [7]. This can result in substantial delays in their initial presentation [8]. Upon presentation, clinicians should be willing to discuss the possibility of a unifying explanation for the signs that others may have noticed. Perceptual agnosia is not limited to the motor domain and can occur in the non-motor domains as well, since it is not uncommon for patients to have a lack of awareness of olfactory dysfunction too [9].

The notion of earlier diagnosis or detection of PD in the context of a disease-modifying or neuroprotective intervention is easy to promote. However, given the current absence of such interventions, the more relevant issue is what makes a timely diagnosis for patients. Giving a diagnosis before any decline in quality of life would arguably not be in most patient’s interest and could instead be detrimental [10]. On the other hand, for individuals with prominent early non-motor symptoms, a timely diagnosis may avoid mis-management and clinicians should be familiar with these features and their treatment [11].

## Historic clinical descriptions

Kinnier Wilson was one of the first clinicians to introduce the concept of symptoms occurring in the pre-diagnostic phase of PD (Kinnier Wilson, Neurology; Volume II, 1940). He described the difficulties in detecting the early motor signs in older people, where stiffness and slowness could be misinterpreted as features of senility. He noted, as did James Parkinson, the insidious nature of these symptoms, such that patients often struggle to recall the onset. He suggested that slowness and bradykinesia are difficult for the patient to detect, but that they frequently precede tremor.

Jean-Martin Charcot had already distinguished bradykinesia as a disabling early clinical feature of PD in many cases (Charcot 1872)—“Long before rigidity actually develops, patients have significant difficulty performing ordinary activities […] even a cursory exam demonstrates that their problem relates more to slowness in the execution of movement rather than a real weakness”. Separately, William Gowers reported that intermittent tremor could occur years before diagnosis (Gowers 1888).

In 1992, Lees described non-specific and sometimes transient symptoms over a 12-year period before the football player Ray Kennedy was eventually diagnosed with PD [8]. In this case report, a range of videos recorded from football matches were reviewed, and it was observed that during matches, Kennedy’s arm was held stiffly and flexed at the elbow. This case report together with the finding that nigral cell loss probably began at least 5 years before bradykinesia was detectable [3], led to a renewed interest in Kinnier Wilson’s Parkinson prodrome.

## Evidence from observational studies

### Clinical cohorts

#### Healthcare records studies

Despite several methodological drawbacks to retrospective studies, the use of routinely collected medical record data offers the advantage of studying large numbers of individuals. Gonera and colleagues were one of the first groups to use this approach in the context of PD [12]. After reviewing the medical records of Dutch patients in general practice, over a decade prior to PD diagnosis, they suggested that prodromal symptoms preceding the diagnosis of PD begin up to 6 years before. These findings were in concordance with the suggested duration of the presymptomatic phase between neuronal loss and the classical syndrome of PD in post-mortem studies [3].

Out of 11 million people registered in The UK Health Improvement Network (THIN) database, 8166 PD patients were compared with 46,455 healthy controls [13]. In addition to the now-recognised early non-motor features, medical codes from clinical consultations were extracted to quantify pre-diagnostic motor markers (symptoms and signs). Tremor was the most common motor marker occurring for the first time up to 10 years prior to diagnosis, but it was not possible to say whether this was a postural/kinetic or rest tremor. Balance problems and rigidity were other early motor symptoms. Subsequently, the combination of motor and non-motor features was used to create a multivariate model to derive a 5-year likelihood ratio for later diagnosis of PD [14].

#### Population-based cohorts

The Rotterdam study is one of the longest running longitudinal studies of PD with 15.8 years of follow-up [15]. Motor assessments came from standardised clinical interviews and assessments carried out by trained nurses. Subjective complaints were extracted from clinical interviews, including stiffness, slowness, tremor, loss of balance, and number of falls in the previous year. Bradykinetic features and tremor tended to be the earliest motor markers, occurring 7 years and 6 years before the diagnosis respectively. In contrast, postural features and changes in gait were reported later (3.8 years before diagnosis) [15].

The TREND cohort study (Tubinger evaluation of Risk factors for Early detection of NeuroDegeneration) recruited 1046 subjects from the general population. After the first screening, individuals with selected non-motor prodromal markers (depression, anxiety, and suggestive RBD symptoms) were followed up. Motor assessment was based on the motor score of Unified Parkinson’s Disease Rating Scale (UPDRS) and a list of five potential motor features beyond general bradykinesia and rest tremor: sialorrhoea, hypophonia, micrographia, arm swing reduction, and dysarthria. No clear differences were found in the motor features occurring in the three different non-motor sub-groups. However, a positive relationship between the motor score of the UPDRS and the number of non-motor prodromal markers was seen [16].

In the Bruneck Study, a group of elderly people were assessed at baseline and at 5-year follow-up [17]. Subtle motor signs defined by Movement Disorders Society (MDS)-research criteria using the UPDRS were used as the study’s endpoint. Objectively reduced sense of smell and hyperechogenicity of the substantia nigra on transcranial sonography were linked to subtle motor signs.

Buchman et al. followed up a large group of people without PD [18]. At post-mortem, nigral neuronal loss and the presence of Lewy body pathology were correlated with the presence of subtle motor signs that had been evaluated with an adapted version of UPDRS. An association was found only with nigral neuronal loss, but not with Lewy body pathology, suggesting that the captured motor signs may be more associated with pathological ageing rather than being PD-specific. Similar results were demonstrated in another post-mortem study in elderly people presenting with mild parkinsonian signs and no diagnosis of PD [19].

The PREDICT-PD study looked for subtle motor signs in higher and lower risk participants [20]. Risks scores were estimated based on the previous identified prodromal markers and risk factors [21]. All the examinations were recorded on video and rated using the MDS-UPDRS together with a subjective global impression by two blinded experts. Alongside raw motor MDS-UPDRS scores, two other clinical scales for mild parkinsonian signs were calculated using the Louis et al. and Berg et al. definitions [22, 23]. The higher risk group had worse scores across all scales compared with the lower risk group, with subtle motor changes being present in 18–31% in the higher risk group and a higher percentage of individuals with a global clinical impression of mild parkinsonism [20].

### “AT-RISK” Groups

#### REM-sleep behaviour disorder

At least ten prospective studies have demonstrated a strong link between idiopathic/isolated RBD and PD, as well as some forms of atypical parkinsonism. Mild associated motor impairment is the strongest predictive marker of future parkinsonism in patients with RBD. In the largest study to date that included 1280 patients from different sleep disorder centres, motor dysfunction was identified using the Purdue Pegboard, alternate-tapping test and timed up-and-go test, along with the motor scale of MDS-UPDRS [24]. The average length of follow-up was 3.6 years, with a maximum of 19 years. Out of 21 potential parameters of phenoconversion, motor impairment was associated with the largest hazard ratio (HR: 3) compared to other clinical and neuroimaging markers. Motor dysfunction was detected 6–9 years before PD diagnosis, with voice changes and facial akinesia being reported as the earliest signs (9.8 years), followed by impairment on the Purdue Pegboard and alternating tapping tests (8.6 and 8.2 years respectively) [25].

Subtle gait abnormalities and balance problems have also been studied in patients with RBD. For example, gait patterns were analysed using a sensor carpet (but no body sensors so arm swing could not been assessed) in 24 polysomnography-confirmed RBD patients and 14 controls [26]. During dual tasking (counting backwards, naming as many animals as possible, subtracting away 7 from 100), RBD patients increased the variability in their step width, and demonstrated more asymmetry in step length when asked to walk quickly.

#### Idiopathic anosmia

Only a few small studies of people with confirmed idiopathic anosmia have been published. Most rely on an empirical definition of hyposmia based on cut-off points on odour identification tests such as University of Pennsylvania Smell Identification Test (UPSIT) or ‘Sniffin sticks’, with alternative explanations for impaired olfaction such as nasal sinus disease or head trauma being ruled out.

The PARS study (Parkinson Associated Risk Study), in which sense of smell was assessed using a self-administered UPSIT, found a similar proportion of subtle motor symptoms (defined by 2 or more symptoms on the Symptom Rating Scale) and average UPDRS motor scores in the normosmic and hyposmic groups [27]. Dopamine transporter (DaT) SPECT imaging was used as the main outcome in this study, but subtle motor symptoms at baseline were not associated with presynaptic dopaminergic degeneration. On the other hand, male gender and constipation, together with hyposmia, were associated with reduced dopaminergic uptake. In the follow-up phase of the PARS study, patients who ‘converted’ to PD had higher UPDRS motor scores than the ‘non-converters’ at baseline [28].

#### Mutation carriers

Glucocerebrosidase (*GBA*) and leucine-rich repeat kinase 2 (*LRRK2*) mutation carriers have a higher age-specific risk of PD diagnosis compared with non-carriers (both estimated to be about 30% at the age of 80 years) [29–31] .

The prevalence of the *LRRK2* G2019S mutation is approximately 1% and 4% of sporadic and familial forms of PD respectively [32]. *LRRK2*-related parkinsonism and idiopathic PD have similar clinical features and response to dopaminergic therapy [33]. However, subtle differences may exist with lower overall motor UPDRS scores, a higher proportion with postural instability and action tremor observed among *LRRK2* mutation carriers with PD [34, 35]. Motor signs assessed using the UPDRS appear to be greater in non-manifesting carriers of *LRRK2* than in non-carriers, and also in first-degree relatives of *LRRK2* PD cases, independently of their mutation status [36, 37]. Using objective gait analysis, carriers show greater variability in gait under challenging conditions and more obvious arm swing asymmetry than controls [38, 39].

In a longitudinal study, whereas motor UPDRS score increased over a 4-year period in carriers of *LRRK2* mutations, the ones who ‘converted’ to PD did not have significantly higher UPDRS motor scores than ‘non-converters’ at baseline [40]. Another longitudinal study followed a group of carriers of *LRRK2* mutations and healthy controls for 5 years [41]. The majority of individuals who fulfilled the MDS research criteria for probable PD at baseline were carriers of *LRRK2* mutations, and during follow-up, the ten patients who were diagnosed with PD were all carriers [23].

Disease-associated *GBA* heterozygous variants are common in PD and are found in 5–10% of patients [42]. Certain mutations in the homozygous state are recognised for causing Gaucher disease [43]. In a longitudinal study of *GBA* homozygous and heterozygous carriers, the UPDRS motor score showed greater progression during 6 years of follow-up in carriers compared to controls [44]. Similarly, two other studies compared controls with Gaucher disease patients and carriers showing that carriers had a higher score in the motor UPDRS than the other groups [45, 46].

## Defining motor prodromes

### Bradykinesia

Bradykinesia is the only physical sign that is present in every patient with PD, using current definitions, and the only cardinal sign that has been correlated with cell loss in the pars compacta of the substantia nigra. Bradykinesia is related to a failure in the recruitment of cortical motor neurons during an intended task [47]. The more complex the action is (i.e., performing different actions simultaneously), the easier it will be to detect bradykinesia.

It is often described by patients as clumsiness or weakness when carrying out delicate tasks [8]. Questions should be directed towards loss of dexterity in repetitive manual tasks such as buttoning clothes, shaving, beating eggs, shampooing, stirring, or writing, and when possible close family members should also be asked whether they have noticed any subtle changes (Table 1).

**Table 1:** Symptoms first noticed by patients

Handwriting changes: progressively smaller, cramped, sloping
Dry eyes due to reduced blinking
Lack of facial expression: distracted, vacant, blank (often reported by relatives and friends)
Lack of arm swing (reported by relatives and friends)
Frozen shoulder
Lack of manual dexterity in repetitive tasks: beating eggs, shaving, typing, playing an instrument
Painful abnormal posture in their foot (typically in young-onset PD)
Scuffing the sole or heel of one foot when walking
Feeling of imbalance

### Tremor

Beyond recognising that tremor may be transient and predate the other features of PD, William Gowers made another key observation—‘In the early stage, only an apparent intermission of contraction is recognised, either in rest or on movement’ (Gowers 1888) [48]. This intermission before the emergence of tremor is readily recognisable in clinical practice and helps to distinguish a postural tremor in PD from that seen in familial essential tremor.

A monosymptomatic rest tremor without bradykinesia has been associated with dopamine denervation on dopamine transporter imaging in some patients and all of these cases go on to develop PD. Other studies have indicated that longstanding asymmetrical postural tremor could be a predictive factor for development of PD and others have argued that even bilateral essential tremor could be a risk factor for PD [49, 50]. Of note, a significant number of patients with ‘benign tremulous Parkinsonism’ have a family history of tremor and/or PD [51, 52].

Neuropathological studies comparing patients with confirmed PD and those with a slowly progressive tremor-dominant parkinsonism suggest that there is less nigral cell loss in the latter [53]. The authors discussed the importance of follow-up for elderly patients with late onset of essential tremor, since parkinsonism may emerge ultimately [53].

### Posture

A stiff-arm held flexed at elbow and the fingers in a flexed-adduction position are tell-tale signs for early parkinsonism. Patients can also appear preternaturally still; maintaining the same position for long periods without the small fidgets and adjustments that healthy people make when sitting or standing (Kinnier Wilson, 1940). In young-onset PD, bradykinesia is not infrequently preceded by foot dystonia and occasionally writer’s cramp by several years. A few patients present with a motor restlessness related to difficulty finding a comfortable posture in which to rest.

### Gait

Walking is an automated, rhythmic motor task that requires both motor and executive skills [54, 55]. The gait pattern is defined by arm swing (amplitude and symmetry), stride (length, off-ground elevation), and coordination between four limbs (rhythm and smoothness). In the elderly, musculoskeletal disorders and the motor signs of diffuse cerebrovascular disease need to be distinguished from true parkinsonism.

Step-to-step variability, decreased amplitude of arm swing or arm swing asymmetry, and reduction in the ‘smoothness’ of gait may be early signs of a Parkinsonian syndrome [56–58].

Several approaches to quantification of gait analysis have been used, from the “timed up-and-go” test, to more sophisticated analysis using sensors. The “timed up-and-go” test is a simple test which has been used to measure mobility and risk of falls in PD and elderly people [59]. However, its applicability as an early motor marker in PD has not been studied in detail.

In the early stages of PD, compensatory mechanisms may prevent gait disturbances. Dual tasking (i.e., asking the patient to do mental arithmetic while walking) is a useful clinical method to unmask subtle signs [60, 61]. This strategy has been used to study people at risk of developing PD, such as non-manifesting carriers of *LRRK2* mutations and people with hyperechogenicity of the substantia nigra. The most characteristic gait patterns found in the at-risk groups under challenging conditions were: higher stride time variability, arm swing asymmetry, and decrease in the smoothness of trunk rotation [38, 57, 60]. Reduction in the smoothness in axial swaying could be due to an increase in axial rigidity, such as that which happens with limb rigidity during distraction tasks (i.e., Froment´s manoeuvre).

Since the prevalence of PD increases with age and gait abnormalities are common in the elderly population, it is important to consider the effects of aging as a confounding factor and consider multifactorial effects of aging on gait from those of PD [62]. Mirelman and colleagues studied 60 healthy controls aged between 30 and 77 years. Whereas arm swing amplitude decreased with age and a dual task, arm swing asymmetry and limb coordination (stepping consistency and rhythm) appeared to be less susceptible to aging [63].

### Handwriting and typing

Handwriting is a fine motor task that imposes a substantial demand on cognition [64]. The concept of handwriting difficulties as an early motor feature of PD dates back to 1817 when James Parkinson documented handwriting changes preceding impairment in walking. Schwab demonstrated that micrographia could be present 4 years earlier than PD diagnosis by reviewing serial signatures from cheque stubs of patients prior to them developing the classical features of PD [65].

Micrographia is defined by gradual reduction in letter size and has been proposed by some as a relevant clinical biomarker [66]. The script is often crabby and cramped, and in right handers tends to slope upwards.

In recent years, with the exploration of technologies to measure objectively handwriting abnormalities in parkinsonism, the term dysgraphia has been introduced to embrace not only script size but other kinetic variables such as fluency, velocity, and duration. This may offer the possibility of picking up more abnormalities in the script of people with PD in an early stage [64]. It is also of note that there is very little correlation between micrographia and bradykinesia severity in PD [67].

The kinematics of typing have also been tracked remotely in de novo patients with PD from the initiation of dopaminergic treatment [68]. The investigators from this study observed that improvement in typing kinematic parameters occurred earlier than in motor scores on the UPDRS after commencing treatment.

### Voice

Hypokinetic dysarthria is a characteristic speech pattern in PD, which is defined by hypophonia, lack of voice modulation, poor articulation, hesitations, and stoppages [69]. Although it has been argued that dysarthria may be an early motor sign, voice changes have received little attention [70]. Researchers working on the Oxford Discovery Parkinson’s Cohort have used a smartphone app and machine learning to distinguish patients with PD, RBD, and controls [71]. Voice analysis was part of the test battery which also included balance, gait, finger tapping, reaction time, and tremor assessment. They instructed participants to perform a sustained phonation (“aaah”) as long as they can. Vibration parameters (amplitude and frequency) were analysed to find instabilities of the vocals folds oscillating patterns. Along with tremor, voice was found to be the most distinguishing feature between the three groups, with voice being the most discriminatory factor between RBD and controls. The same team has demonstrated how this approach could be used to predict the milestones of progression in PD [72]. Another study focused on abnormal speech patterns in prodromal PD, showing that voice frequency variability could be detected up to 5 years prior the diagnosis [73].

### Blink rate and facial hypomimia

The standard rate of blinking can range between 14 and 25 blinks per minute. It is influenced by mental tasks (reading, watching a film, and speaking) and also mental state (anxiety and depression). Several studies have proved a positive association between blinking rate and central levels of dopamine by comparing conditions with inverse dopamine activity (PD and schizophrenia) and testing the effect of dopamine agonists and antagonists on eye-blink rate [74].

Relatives and friends often notice a frozen facial expression which they describe as a dullness, a ‘poker face’, ‘a mask’, a sadness, or a coldness. A reduction of blink rate as a feature of PD has long been recognised—‘A valuable early symptom is infrequent blinking of the eyelids’ (Kinnier Wilson 1940). This may lead to a complaint of dry sore eyes or watering of the eyes. However, Fitzpatrick and colleagues could not find any link between blink rate and disease severity and duration in one study, which led to the conclusion that reduced blink rate is an early and unalterable feature of PD [75].

### Subthreshold parkinsonism vs normal aging

Approximately 30–40% of elderly people without a known neurological condition exhibit ‘soft’ basal ganglia (extrapyramidal) signs on clinical examination, including slowness, postural changes, and gait problems [22, 62] (Table 2). Most of these patients do not have early PD and the signs can be attributed to old age, diffuse cerebrovascular disease, and musculoskeletal problems [76, 77].

**Table 2:** Features of ageing as opposed to Parkinsonism

Isolated shuffling broad-based gait
General slowness not particularly affecting dexterity
Quivering tremulous voice
Unsteadiness with falls
No response to levodopa

Vascular disease and risk factors, in particular the combination of diabetes mellitus and heart disease, have been shown to increase the probability of parkinsonian signs by 70% [77]. Bennet and colleagues showed that people with subclinical parkinsonian signs had a risk of mortality twice as high as those without it [62].

Some clues from the examination can be extracted to differentiate normal ageing from PD. The most important one is that in general, the progression of symptoms in PD is quicker than that which occurs due to ageing. Asymmetry between sides, the sequence effect on finger tapping, hypomimia, changes in voice modulation, and the presence of other non-motor symptoms are all helpful in distinguishing the pre-diagnostic stages of PD from the effects of aging [78].

## Methods of identifying subtle motor abnormalities

Motor analysis in the early phases of PD has several limitations. Heterogeneity in prodromal phenotypes may make it difficult to standardise methods of analysis. As mentioned, with clinical rating scales, parkinsonian signs have been described in between 30 and 40% of the elderly population without PD [62]. No consensus exists on the ideal method of measuring motor dysfunction in the early stages of PD, including which scales to use, which kinetic parameter are best to analyse (e.g., amplitude or velocity for bradykinesia), and under what conditions (home monitoring, lab or clinic environment, and challenging conditions). Standardised approaches may be required to set the boundaries between prodromal and established PD [79].

**Fig. 1:**
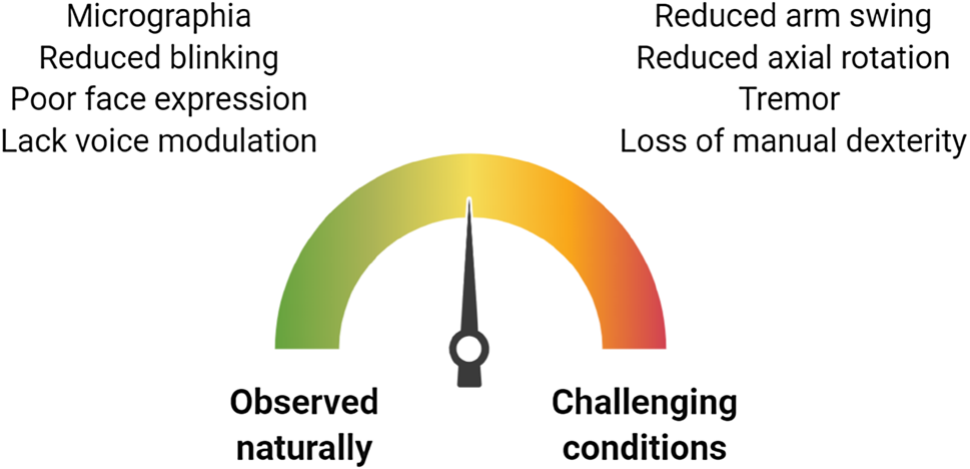
On the left, features to explore on normal condition (spontaneous movements). On the right, other signs emerge under challenging conditions (dual tasking and fast walking)

Extranigral structures may play a compensatory role in the progressive dopamine loss in PD [80]. At early stages, such mechanisms are strong enough to mask such motor deficits. Thus, as outlined in some of the aforementioned studies, clinical examination and assessment should aim to challenge compensatory mechanisms (Fig. 1) [6].

### Questionnaires

Questionnaires asking about subjective motor complaints have been used as a diagnostic tool with apparent high sensitivity and specificity [81]. However, the role of bias in reporting subjective symptoms has to be considered [82].

Telephone questionnaires have been used to study patients’ perception of prodromal symptoms [83]. In one study, slowing of fine hand movements, general bradykinesia, dysarthria, and reduced arm swing were noticed between 2.2 and 4.7 years before the patient fulfilled the diagnostic criteria of PD. Although PD patients reported motor difficulties more frequently, 10% of age-matched controls had also noticed some changes in their dexterity and speed of movement.

Maraki and colleagues used a motor screening battery, including a questionnaire focused on gait and postural difficulties in elderly people taking part of the HELIAD study (Hellenic Longitudinal Investigation of Aging and Diet cohort). Having at least one subjective gait/postural difficulty increased the probability of having prodromal PD (based on the MDS research criteria) [23]. Difficulty walking outdoors, poor balance, using a walking aid, and the presence of a shuffling gait were reported more commonly (between 25 and 35%) by subjects with probable prodromal PD [84].

### Clinical scales

The standard of motor assessment for established PD is the MDS-UPDRS [23]. It is a semi-quantitative scale based on several motor tasks such as rapid simple movements (finger and toe tapping test) addressed to evaluate bradykinesia.

However, it is important to note that the UPDRS was not designed for use in the prodromal stages of PD, and may not be sensitive enough to pick up subtle motor features at early stages [6]. The MDS research criteria for prodromal PD recommends a cut-off of 6 (overall score) and 3 (excluding action tremor) for defining subthreshold parkinsonism [23]. In one study, bradykinesia-related items presented the lowest reliability of all the UPDRS parameters with a finger-tapping interrater reliability Kappa coefficient below 0.50 [85].

At the prodromal stages of PD, the scoring used in the MDS-UPDRS is susceptible to a floor effect between scores of 0 and 1 (normal and slight abnormalities). To rectify this limitation, a modified bradykinesia score was created which separately score three kinetic parameters (frequency, rhythm, and amplitude) for each movement [86]. However, even with these modifications, there are additional important features such as manual dexterity, posture, and gait under challenging conditions that are not captured.

### Technology

There has been considerable interest in technological solutions to the capture of subtle motor features of PD. However, a reliance on technology, including artificial intelligence along with machine and deep learning, and other ‘big data’ approaches, can lead to oversimplification and loss of appreciation for the particularities and subtleties of the motor features of PD. The clinician’s observations and perception and the evaluation of a patient’s co-morbidities are essential to avoid misdiagnosis.

**Fig. 2:**
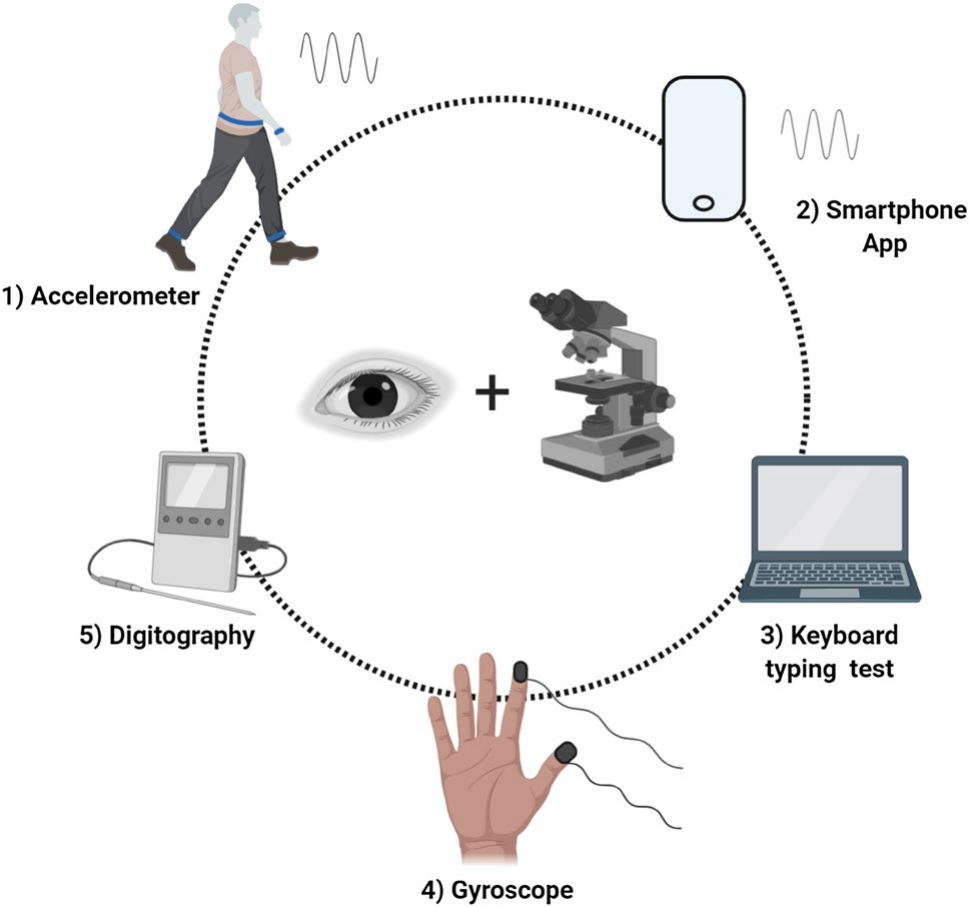
New technology era. The importance of combining clinical expertise with more sophisticated quantitative tools. 1: Body sensors with incorporated accelerometers able to monitor movement in a home-environment. 2: Smartphone-based tools to assess bradykinesia, tremor, and voice modulation. 3: Keyboard typing test to quantify velocity and rhythm during the task. 4: Digital sensors associated with a gyroscope to quantify changes in velocity, amplitude, and rhythm during finger tapping. 5: Digital screen and sensory pen for the handwriting and spiral drawing assessment

Technological approaches can be classified according to whether the assessments are home or lab-based, the type of device (body sensors, smartphone app, 3D motion capture, and computer keyboard), and kind of movement tested (gait, finger-tapping, handwriting, and spiral drawing) (Fig. 2). One important question is whether, particularly in the prodrome, technology should be validated against scales used for established PD, or whether new scoring paradigms should be prioritised [87]. There is now the emerging possibility that ‘big data’ approaches will help define new sub-groups of patients based on both motor and non-motor features and other clinical characteristics, making us less reliant on the traditional scales [88]. While technology will certainly play a far greater role in the assessment and management of PD, it remains hard to envisage how it could wholly replace a detailed neurological examination, appreciation of the complex, and heterogeneous motor and non-motor manifestations of PD and clinical expertise.

## Conclusion

Subtle motor dysfunction is an important but relatively neglected part of the prodromal phase of PD. In contrast to individual non-motor features, by current definition, all patients with PD will develop early motor signs, and motor dysfunction will progress as the disease advances. It is clear that available questionnaires and clinical scales are not suitably adapted for early stages of PD, and electronic measures are not currently sufficiently developed and validated. New ways and measures are still needed to reliably pick up motor dysfunction at the earliest stage.
